# Fully Flexible Covalent Organic Frameworks for Fluorescence Sensing 2,4,6-Trinitrophenol and *p*-Nitrophenol

**DOI:** 10.3390/polym15030653

**Published:** 2023-01-27

**Authors:** Hai Zhu, Tong-Mou Geng, Kai-Bin Tang

**Affiliations:** 1 Department of Chemistry, University of Science and Technology of China, Hefei 230026, China; 2 Anhui Province Key Laboratory of Optoelectronic and Magnetism Functional Materials, School of Chemistry and Chemical Engineering, Anqing Normal University, Anqing 246011, China

**Keywords:** covalent organic frameworks, flexible, fluorescent sense, 4-nitrophenol, 2,4,6-trinitrophenol

## Abstract

Nitrophenols are important nitroaromatic compounds, both important environmental pollutants and dangerous explosives, posing a devastating danger and pollution threat to humans. It is vital to detect efficiently trace nitrophenols in the environment. In this contribution, a series of fully flexible cyclotriphosphazene-based COFs (FFCP COFs: HDADE, HBAPB, and HBPDA), prepared with both a flexible knot and flexible linkers of different lengths, were used for sensing 2,4,6-trinitrophenol (TNP) and *p*-nitrophenol (*p*-NP) in real time with excellent sensitivity and selectivity. The quenching constants of HDADE by TNP, HBAPB, and HBPDA by *p*-NP are 6.29 × 10^4^, 2.17 × 10^5^, and 2.48 × 10^5^ L·mol^–1^, respectively. The LODs of TNP and *p*-NP are 1.19 × 10^−11^, 6.91 × 10^−12^, and 6.05 × 10^−12^ mol·L^−1^. Their sensitivities increase with the linker length, which is better than the corresponding COFs composed of rigid linkers. There is only a photoinduced electron transfer mechanism in the fluorescence quenching of HBPDA by *p*-NP. Meanwhile, the mechanisms of photoinduced charge transfer and resonance energy transfer exist in the fluorescence quenching of HDADE by TNP and the fluorescence quenching of HBAPB by *p*-NP.

## 1. Introduction

Nitrophenols, as a category of phenolic nitroaromatic compounds (NACs), have been in universal use in the intermediates of herbicide, pesticide, dyes, antiseptic, plastics, pharmacy, and other chemicals [1,2]. Because nitrophenols are a class of highly toxic and harmful phenols that can cause serious health damages and public nuisances, they have been considered main contaminants by the Environmental Protection Agency of the United States (EPA) [2,3,4].

*p*-Nitrophenol (*p*-NP) has been one of the most commonly used phenols worldwide for the manufacture of analgesics, dyes, and pesticides and for leather processing, thus attracting attention to public safety and environmental issues [2,3,4,5]. Because of its toxicological and healthy effects, *p*-NP’s lifetime health advisory (LHA) level in drinking water has been identified to be 60 µg L^−1^ [1], and it has been listed as an important toxic contaminant by the EPA [6]. *p*-NP has high stability and solubility in aqueous medium and is one of the most harmful water pollutants. Not only does *p*-NP cause severe environmental problems, but it is also carcinogenic or genotoxic to mankind and wildlife [5].

2,4,6-Trinitrophenol (TNP) is both used as a powerful explosive and one of the environmental pollutants [7,8]. As the most commonly used volatile components, TNP poses a massive threat to humankind due to both fatal effects and the characteristics of toxic pollutants. Among different nitroexplosives, TNP can engender a strong explosive force comparable to trinitrotoluene (TNT) and as a well-known toxic pollutant [9,10]. Several studies have indicated that TNP and its metabolite, picramic acid, have oncogenic, teratogenic, and mutagenic effects [10]. As a result of its strong electron-withdrawing capacity, it is difficult to degrade nontoxic substances [11]. Because TNP has high solubility in water, poor biodegradation, and strong toxicity, it has been ranked at the front ranks of contaminants [8]. In addition, TNP can give rise to severe eye and skin irritation, very serious respiratory disorders, dizziness, liver or kidney injury, mutagenesis, and so on, that poses a significant threat to mankind’s health. However, the continual application of TNP in dyestuff, fireworks, textile, and leather industries inevitably causes its release into the surroundings during manufacture and use [10]. Thus, the accurate and ultrasensitive detection of *p*-NP and TNP is important for pollution control [2,4,8,11].

The traditional detection methods currently reported for trace *p*-NP and DNP mainly consist of spectrophotometry, high-performance liquid chromatography (HPLC), surface-enhanced Raman spectroscopy, electrophoresis, gas chromatography-mass spectroscopy (GC-MS), mass spectrometry, and electrochemical methods [1,6]. Among them, fluorescence sensing based on various fluorescent materials offers several advantages, such as excellent sensitivity, simplicity, stability, rapid response time, and low cost [10,12], for the determination of NACs. Due to the existence of electron-deficient nitrogroups, most NACs can be suitable as fluorescent quenchers, which makes fluorescence quenching-based sensors the most effective tools for fluorescence-detecting NACs whether in the liquid phase or in the solid phase [13,14].

The covalent organic frameworks (COFs) are emerging porous organic materials consisting of strong covalent bond connections between light elements (C, H, N, O, B). As one of the essential components of porous organic polymer (POP) in long-range ordered crystalline states, COFs have attracted widespread attention and developed fleetly since COFs were first discovered by Yaghi et al. in 2005 [15]. Their high porosity, good crystal morphology, and robust functionality give COPs excellent development potential [16]. Due to the poor stability of boroxane and borate, the COFs prepared from aldehyde-amine Schiff base reactions show sufficiently high heat endurance and chemical stabilities, which are studied in fluorescence sensing applications [16,17,18,19,20,21,22,23]. The COFs have been used in fluorescence-sensing TNP [24,25,26,27,28,29,30]. However, reports of COFs fluorescence-sensing *p*-NP are relatively rare [5,28].

COFs, like conjugated microporous polymers (CMPs), and their building blocks are predominantly conjugated rigid units. Most COF structures containing rigid C=N bonds, while giving fixed-size regular pores and channels, cannot optimize the superiorities of both an active site and a functional group. To make full use of regular pores and a material uniform position in practice, a few flexible COFs are synthesized with the merits of elastic and adaptive ability [31,32,33]. Most of the COFs used for fluorescence sensing were constructed from rigid building blocks, and only a few COFs’ built-in flexible knots were used for fluorescence sensing [5,34]. Although these flexible COFs are made from flexible knots, the building blocks building their linker are mostly a rigid [35,36,37] or semiflexible motif [38]. In this study, fully flexible cyclophosphazene-based COFs (FFCP COFs) constructed entirely of flexible building blocks (HDADE, HBAPB, and HBPDA) were synthesized by Schiff base polymerization reactions and via the solvent thermal method with flexible hexa(4-formyl-phenoxy) cyclotriphosphazene (NOP-6-CHO) as a knot and flexible 4,4′-diaminodiphenyl ether (DADE), 1,4-bis(4-aminophenoxy)benzene (BAPB), and 4,4′-(1,1′-biphenyl-4,4′-diyldioxy)dianiline (BPDA) as linkers.

## 2. Materials and Methods

### 2.1. Materials

FFCP COFs, HDADE, HBAPB, and HBPDA were synthesized, respectively with NOP-6-CHO- as a node, DADE, BAPB, and BPDA as linkers in our previous work [39] and are detailed in Section S1 [40]. *p*-NP, TNP, and other starting materials and chemicals were bought from Aladdin Co., Shanghai, China.

### 2.2. Methods

UV-VIS spectra were carried through on a Shimadzu UV-2501PC instrument. Fluorescence spectra were recorded on an LS55 spectrophotometer (PerkinElmer). IR spectral analyses were performed on KBr pellets using an iS50 FTIR instrument. Solid-state ^13^C NMR spectra of COFs were kept an account of by a Bruker Digital Avance III HD 400 WB (400 MHz) NMR spectrometer. Elemental analyses were performed on a Vario EL III CHN cube (C,H,N,S) elemental analyzer. TGA was performed with a CDR-4P TGA Thermal Analyzer under a N_2_ atmosphere from room temperature to 800 °C with a heating rate of 10 °C min^−1^. Powder X-ray diffraction (PXRD) analyses of COFs were performed on a Bruker AXS D8 Discover X-ray diffractometer in a 2θ range from 1.5° to 40°. The surface morphology of the materials was analyzed by scanning an electron microscope (SEM) on a S-3400 N microscope instrument. All the COFs were degassed at 100 °C for 6 h under vacuum before the analysis. The surface areas of the COFs were calculated with the Brunauer-Emmett-Teller (BET) and Langmuir model in the Rouquerol plots. The pore size distributions of the COFs were obtained from N_2_ isotherms in accordance with the nonlocal density functional theory (NLDFT) method.

### 2.3. Fluorescence Spectra

#### 2.3.1. Solid State Fluorescence

The COF material is fully ground and carefully loaded into the small device (custom metal device, one-side light transmission). Wipe the small device around and put it into the fluorescence spectrophotometer for testing the solid state fluorescence spectrum. First, set an excitation wavelength λ_ex1_. obtain the emission peak position λ_em1_ and then use the emission wavelength λ_em1_. In turn, the wavelength λ_ex2_ of the excitation peak was as excitation wavelength for doing emission spectrum. This is repeated until λ_em_ is obtained using the excitation wavelength λ_ex_, which is consistent with λex obtained using the emission wavelength λem. The final test yielded the solid-state excitation and emission spectra of the COF material.

#### 2.3.2. Fluorescence Quenching Experiments

The fluorescence quenching conditions, such as the solvent, polymer concentration, excitation wavelength, scanning speed, slit width, and response time, were optimized, after which the fluorescence quenching experiments were performed. The fluorescence spectrum was measured by taking 2 mL of COF dispersion in a cuvette at a certain excitation wavelength, slit width and scanning speed, and the fluorescence intensity at the maximum absorption wavelength in the emission spectrum was taken as I_0_. Gradually add the analyte, and take its fluorescence intensity at the maximum wavelength as I. The analyte solution volumes are added not more than 100 μL to avoid errors. The I_0_/I and analyte concentration curve was made, and the slope of the straight line part is the quenching constant.

#### 2.3.3. Fluorescence Stability Experiments

An amount of 25 mg of HDADE, HBAPB, or HBPDA was dispersed in 25 mL ACN, THF, or DOX in a volumetric flask and stirred overnight to form well-distributed dispersion. Then, 2 mL dispersion was taken out into a cuvette to make a fluorescent measurement. After the measurement, the dispersion was carefully retrieved to the volumetric flask, and 18.75 μL or 12.5 μL TNP or *p*-NP solution (0.1 mol L^−1^) was added into the volumetric flask to make the concentration of TNP or *p*-NP in the system 7.5 × 10^−5^, 5.0 × 10^−5^ mol L^−1^, or 7.5 × 10^−5^ mol L^−1^. The volumetric flask was shaken to make sure that the whole dispersion mixed well. Then, 2 mL dispersion was taken out into a cuvette to measure its fluorescent spectrum. That is the first cycling test. Afterward, the dispersion was retrieved. The whole dispersion was centrifuged, and the residual solid was washed successively with hot ethanol to remove the guest molecules of TNP or *p*-NP completely. The regenerated HDADE, HBAPB, or HBPDA was dispersed in 25 mL ACN, THF, or DOX in the volumetric flask to repeat the second cycling test. In the cycling test, it is important to make sure that the concentration of dispersion used for conducting the fluorescent spectrum was at the same level (1 mg mL^−1^), so we amplified the amount of the sample in this procedure to reduce the experimental error.

## 3. Results and Discussion

### 3.1. Structural Characterization

The structures of the obtained FFCP COFs were analyzed and characterized using Fourier-transform infrared spectra (FTIR, Figure S1), solid state nuclear magnetic resonance spectroscopy (ss ^13^C NMR, Figure S2), and UV–VIS spectroscopy (Figure S3). Their chemical compositions were tested via elemental analysis (S-2). The results are shown to be the target products.^39^ The elemental analysis of HDADE and HBAPB showed that the contents of H were inconsistent experimental and theoretical values, which is a common phenomenon in porous organic polymers. It is because of the error caused by calculating the elemental contents by not considering the unreacted end groups or caused by inadequate combustion during elemental analysis. UV–VIS spectra indicated that the FFCP COFs extended the conjugated properties on their frameworks. However, the conjugacy of the FFCP COFs was lower than that of rigid-linker COFs [5,36,39,41]. With the increase in linker length, the conjugacy of FFCP COFs decreases.

The morphology of the synthesized FFCP COFs was analyzed by scanning electron microscopy (SEM, Figure S4) and X-ray diffraction (XRD). The crystal structures of the FFCP COFs are imitated with the Materials Studio (MS) software (Figure S5). The FFCP COFs are spherical structures [36,42]. HDADE, HBAPB, and HBPDA had certain crystallization [32,43]. The crystallinity increased with the linker length and was better than that of the rigid-linker COFs [5,36,41]. The FFCP COFs were microporous materials. The BET-specific surface areas of HDADE, HBAPB, and HBPDA were severally 790, 975, and 1165 m^2^·g^−1^, which were higher than those of the rigid-linker COFs (Figure S6 and Table S1) [5,36]. With the linker length increased, so did the specific surface area. According to the pore size distribution curves near the micropore, the micropores of HDADE, HBAPB, and HBPDA were severally focused in 0.690, 0.691, and 0.688 nm (Figure S6b). The mean pore sizes of HDADE, HBAPB, and HBPDA were 4.03, 2.69, and 2.86 nm, respectively. The pore size of the COFs prepared from a rigid linker increased with the length of the linkers [5,36,39,41]. Nevertheless, there is no such orderliness in pore size in Figure S6 and Table S1, which may be due to the self-regulating flexible units. The obtained FFCP COFs were chemical (Figure S7) and thermal stability with decomposition temperatures of 446/412, 438/412, and 445/410 °C in the nitrogen/air atmosphere (Figures S8 and S9), which indicated that the flexible linker length had little effect on the thermal stability of the FFCP COFs and was similar to the COFs with rigid units [5,36,41].

### 3.2. Effect of Solvent on the Fluorescence Performance of the FFCP COFs

**Figure 1:**
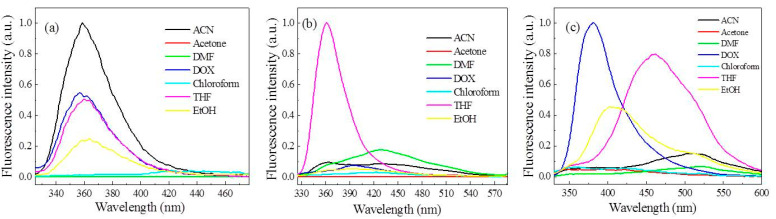
Fluorescence emission spectra of the FFCP COFs dispersed in different organic solvents: (**a**) HDADE (excited at 248 nm), (**b**) HBAPB (excited at 305 nm), and (**c**) HBPDA (excited at 311 nm).

We investigated the effects of different solvents (e.g., acetone, 1,4-dioxane (DOX), *N*,*N*-dimethylformamide (DMF), acetonitrile (ACN), tetrahydrofuran (THF), ethanol (EtOH), and chloroform) on the fluorescence performance of the FFCP COFs (1.0 mg mL^−1^) at room temperature [44]. For HDADE, the ACN dispersion of HDADE has the most vigorous fluorescence intensity with a maximum emission peak of 359 nm excited at 248 nm [45,46]. HDADE has moderate fluorescence intensities in THF, DOX, and ethanol dispersion, and has weak fluorescence in other solvent dispersions, such as acetone, chloroform, and DMF. Under excitation at 305 nm, HBAPB emitted the most robust fluorescence with a maximum emission wavelength of 361 nm scattered in THF, whereas HBAPB emitted low fluorescence emission intensities scattered in other solvents. When excited with 311 nm wavelength, HBPDA showed the most robust fluorescence emission spectrum in DOX dispersion with a maximum emission wavelength of 382 nm. HBPDA in THF and ethanol also had high fluorescence emission and demonstrated significant redshifts of 461 (THF) and 408 (ethanol) nm, respectively (Figure 1). These exciting results suggest that HDADE, HBAPB, and HBPDA can be efficiently used as fluorescent sensors to detect small molecules in solvents, such as ACN, THF, or DOX [47,48,49,50]. This may be due to the much greater enthalpy of HDADE interacting with ACN, HBAPB with THF, or HBPDA with DOX than other solvents. Due to the strong solvation between them, the HDADE network in ACN, the HBAPB network in THF, or the HBPDA network in DOX is rigid, extending the chain more. The nonradiative energy loss caused by the flexibility of bond rotation is minimal. Thus, the intrinsic fluorescent HDADE, HBAPB, or HBPDA powders achieve more robust luminescence properties in the ACN, THF, or DOX liquid phase [51,52,53,54,55].

### 3.3. Response Time

**Figure 2:**
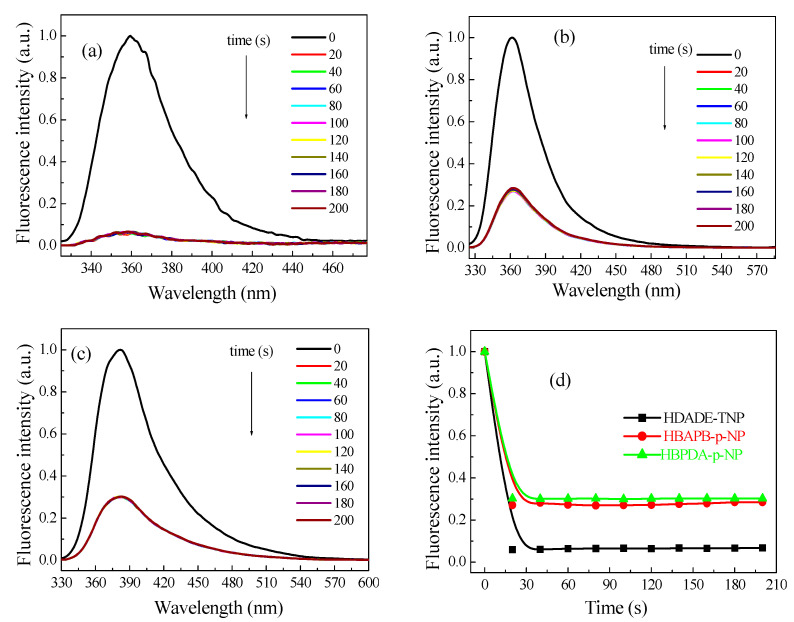
Fluorescence intensities of the FFCP COFs upon addition of TNP or *p*-NP for different periods of time. (**a**) HDADE dispersed in ACN (excited at 248 nm, TNP = 7.5 × 10^−5^ mol L^−1^), (**b**) HBAPB dispersed in THF (excited at 305 nm, *p*-NP = 5.0 × 10^−5^ mol L^−1^, (**c**) HBPDA dispersed in DOX (excited at 311 nm, *p*-NP = 7.5 × 10^−5^ mol L^−1^), (**d**) the plots of fluorescence maxima of the FFCP COFs as the function of time.

Figure 2 shows that 7.5 × 10^−5^ mol L^−1^ TNP was mixed with 1.0 mg mL^−1^ ACN dispersion of HDADE, or 5.0 × 10^−5^ mol L^−1^ *p*-NP was mixed with 1.0 mg mL^−1^ THF dispersion of HBAPB, or 7.5 × 10^−5^ mol L^−1^ *p*-NP was mixed with DOX dispersion of HBPDA; their fluorescence was immediately quenched. The initial fluorescence intensities decreased substantially over time and remained unchanged after that [55]. It is shown that the obtained FFCP COFs can detect TNP or *p*-NP in real time [5,56]. The instant responses of FFCP COFs to TNP and *p*-NP are because the π electrons can move rapidly in the conjugated networks, and the porous network structures allow TNP and *p*-NP to diffuse rapidly and approach the action sites on the networks [5,41].

### 3.4. Sensitivity of the FFCP COFs

**Figure 3:**
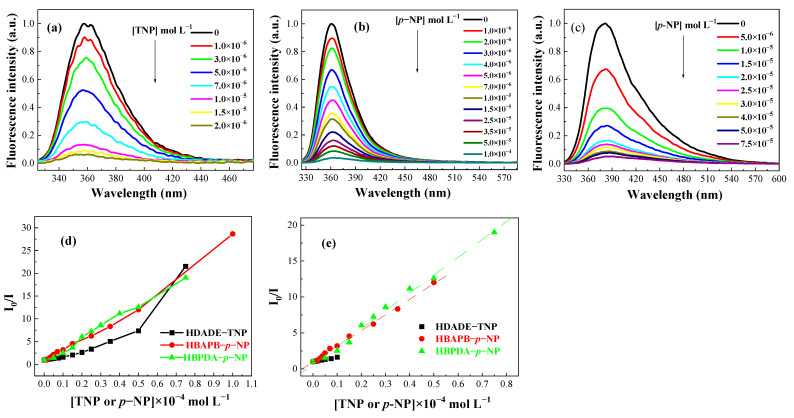
The diversifications of fluorescence intensities of the FFCP COFs: (**a**) HDADE dispersed in ACN (excited at 248 nm), (**b**) HBAPB dispersed in THF (excited at 305 nm), (**c**) HBPDA dispersed in DOX (excited at 311 nm). (**d**) Relative fluorescence intensity (I_0_/I) in dispersions adding TNP or *p*-NP; (**e**) Stern-Volmer plots of the FFCP COFs.

We tested the chemosensing properties of HDADE, HBAPB, and HBPDA dispersions to various nitroaromatic compounds (NACs). These NACs include *p*-NP, TNP, 2,4-dinitrotoluene (DNT), *m*-nitrophenol (*m*-NP), 2,4-dinitrophenol (DNP), *2*-nitrophenol (*o*-NP), para-dinitrobenzene (*p*-DNB), nitrobenzene (NB), *p*-nitrotoluene (*p*-NT), and *m*-dinitrobenzene (*m*-DNB). The degree of reduction in the fluorescence intensity was related to the property of the NACs [56]. In these NACs, TNP significantly quenched the fluorescence of HDADE, while *p*-NP quenched the fluorescence of HBAPB and HBPDA. The fluorescence intensities continued to decrease as the concentrations of TNP or *p*-NP increased (Figure 3). The quenching constants (K_SV_) of the TNP for HDADE, *p*-NP for HBAPB, and HBPDA were calculated from Stern-Volmer equations to be severally 6.29 × 10^4^, 2.17 × 10^5^, and 2.48 × 10^5^ L mol^−1^, was similar to the other COF-based sensors (Table 1 and Table S1) [25,27,57].

**Figure 4:**
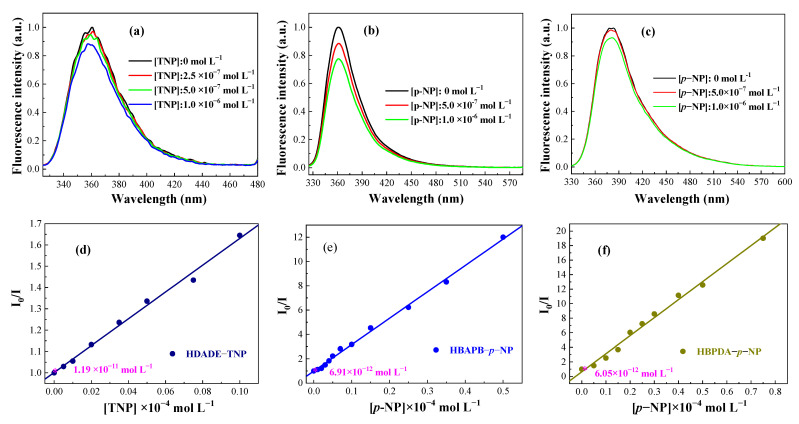
The LODs of the FFCP COFs: (**a**) HDADE dispersed in ACN (excited at 248 nm) for TNP, (**b**) HBAPB dispersed in THF (excited at 305 nm), and (**c**) HBPDA dispersed in DOX (excited at 311 nm) for *p*-NP. The S−V plots of the FFCP COFs: (**d**) HDADE for TNP, (**e**) HBAPB for *p*-NP and (**f**) BPDA.

In addition, according to the definition of the limit of detection (LOD), LOD = 3 S/ρ, where S is the standard deviation and ρ is the slope of the S–V curve, that is, the Ksv value. The LODs for TNP and *p*-NP were 1.19 × 10^−11^, 6.91 × 10^−12^, and 6.05 × 10^−12^ mol L^−1^ (Figure 4 and Table 1), which again indicated that the three FFCP COFs had excellent sensitivity to TNP or *p*-NP, outperforming other COFs′ chemosensors (Table 2) [8,58,59]. This is due to the fact that although the FFCP COFs were poorly conjugated, they contained fluorophores in their skeleton. Moreover, their flexible units had good flexibility and adaptability [31,32,33]. In addition, the FFCP COFs had large specific surface areas, making them have more interaction points with nitrophenol than the COFs of the rigid linker construction [5,36].

### 3.5. Selectivity of the FFCP COFs

**Figure 5:**
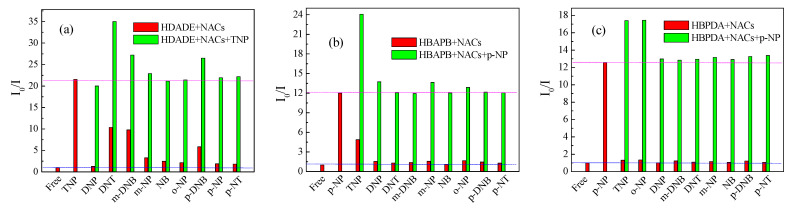
Selectivity and competitivity of the FFCP COFs for sensing TNP or *p*-NP: (**a**) I_0_/I of HDADE added various NACs, followed by TNP (7.5 × 10^−5^ mol L^−1^, excitation wavelength = 248 nm). (**b**) I_0_/I of HBAPB added various NACs, followed by *p*-NP (5.0 × 10^−5^ mol L^−1^, excitation wavelength = 305 nm). (**c**) I_0_/I of HBPDA added various NACs, followed by *p*-NP (7.5 × 10^−5^ mol L^−1^, excitation wavelength = 311 nm).

Adding the same concentration of NACs (7.5 × 10^−5^ mol L^−1^) to the 1.0 mg mL^−1^ ACN dispersion of HDADE, the fluorescence intensity of HDADE was significantly affected by TNP; DNT, *m*-DNB, and *p*-DNB had relatively some impact on the fluorescence intensity of HDADE; and other NACs showed relatively small fluorescence quenching of HDADE, which suggested that HDADE has some fluorescence sensing selectivity for TNP. The addition of the same concentration of NACs (5.0 × 10^−5^ mol L^−1^) to the 1.0 mg mL^−1^ dispersion of THF-HBAPB or DOX dispersion of HBPDA showed that *p*-NP greatly affected the fluorescence intensities of HBAPB and HBPDA, and TNP affected the fluorescence intensity of HBAPB. Other NACs have the relatively small fluorescence quenching of HBAPB and HBPDA, indicating a high fluorescence sensing selectivity for *p*-NP (Figure 5, red column) [49]. To further assess the selectivity of HDADE for TNP, HBAPB, and HBPDA for *p*-NP, competition experimentations were actualized in the existence of the rest of NACs (Figure 5, green column). It can be seen that for HDADE, besides some interference with TNP fluorescence detection by DNT, *m*-DNB, and *p*-DNB, other NACs’ interference with TNP fluorescence detection was negligible. For HBAPB, in addition to TNP interference with *p*-NP, and for HBPDA, except for TNP and *o*-NP interference with *p*-NP, other NACs had little interference with *p*-NP detection [8].

### 3.6. Fluorescence Sensing Mechanism of the FFCP COFs

**Figure 6:**
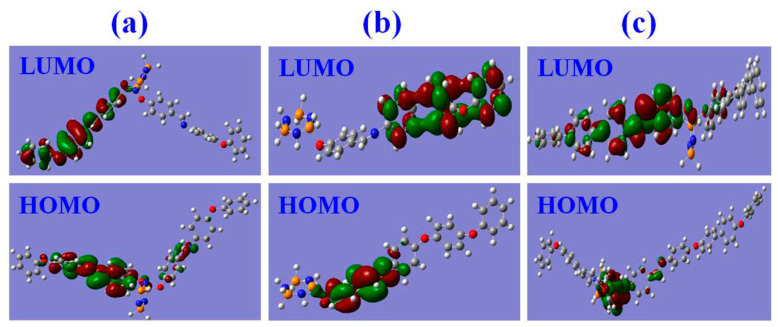
HOMO and LUMO orbital diagrams of the FFCP COFs: (**a**) HDADE, (**b**) HBAPB, and (**c**) HBPDA. The molecular orbital calculations were carried out with the Gaussian 09 D.01 program at the B3LYP/6-31G (d) level.

**Figure 7:**
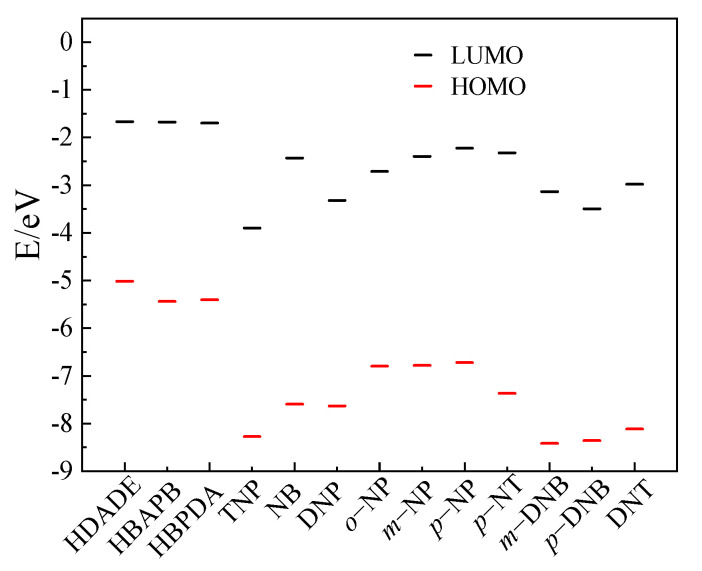
The calculations of HOMO and LUMO energy levels for the FFCP COPs and NACs. All the molecular orbital calculations were implemented with the Gaussian 09 program at the B3LYP/6-31G (d) level.

**Figure 8:**
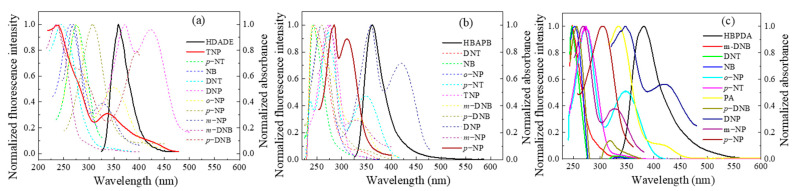
Normalized UV–VIS spectra of the NACs and fluorescence emission spectra of the FFCP COFs: (**a**) HDADE dispersed in ACN (excited at 248 nm), (**b**) HBAPB dispersed in THF (excited at 305 nm), and (**c**) HBPDA dispersed in DOX (excited at 311 nm).

To elucidate the mechanisms of the FFCP COFs’ fluorescence quenching by TNP or *p*-NP, we compared the orbital energy levels of the lowest unoccupied molecular orbital (LUMO) and the highest occupied molecular orbital (HOMO) of the FFCP COFs and NACs (Figure 6 and Figure 7 and Table S2). The LUMO energy levels of the FFCP COFs were above the LUMO levels of the NACs, which supplied the driving force for the devolving of electrons from the FFCP COFs to the NACs, resulting in the fluorescence quenching of the FFCP COFs [25,26]. In addition, the fluorescence spectra of the FFCP COFs and the electron absorption spectra of the NACs revealed that the fluorescence spectrum of HDADE largely overlays with the UV–VIS spectrum of TNP, and that the fluorescence emission spectrum of HBAPB also partially overlaps with the UV-VIS spectrum of *p*-NP, which indicated that there is energy transfer occurring between HDADE and TNP and between HBAPB and *p*-NP, thus enhancing the fluorescent quenching effect [5,25]. Thus, the fluorescent quenching process of HDADE by TNP and *p*-NP to HBAPB has both photoinduced charge transfer and resonance energy transfer procedures [26]. However, there was little spectral overlap between the UV–VIS spectra of *p*-NP and the fluorescent emission spectrum of HBPDA, meaning that the HBPDA fluorescent quenching process by *p*-NP is only a photoinduced electron transfer process (Figure 8). After five reuses, the fluorescence intensities of the FFCP COFs were not significantly reduced (Figure S10), and their FTIR and PXRD patterns were not significantly altered (Figures S11 and S12), indicating the excellent fluorescence stability and chemical stability [25,55].

## 4. Conclusions

In summary, through a Schiff base polymerization reaction and solvothermal method, FFCP COFs completely composed of flexible building blocks (HDADE, HBAPB, and HBPDA) were synthesized with flexible NOP-6-CHO as the junction and flexible DADE, BAPB, and BPDA as the connecting agent. FFCP COFs have high thermal and chemical stability and are microporous materials of the crystalline structure. HDADE, HBAPB, and HBPDA exhibit excellent fluorescence performance in some organic solvent dispersions. HDADE, HBAPB, and HBPDA can sense TNP and *p*-NP in real time with excellent sensitivity and selectivity. The quenching constants of HDADE by TNP, HBAPB, and HBPDA by *p*-NP are 6.29 × 10^4^, 2.17 × 10^5^, and 2.48 × 10^5^ L·mol^–1^, respectively. The LODs of TNP and *p*-NP are 1.19 × 10^−11^, 6.91 × 10^−12^, and 6.05 × 10^−12^ mol·L^−1^. The sensitivity increases with the linker length and is higher than that of the COFs composed of rigid units. There is only a photoinduced energy transfer mechanism in the fluorescent quenching of HBPDA by *p*-NP. Meanwhile, there are both photoinduced energy transfer and resonance energy transfer mechanisms in both the fluorescence quenching of HDADE by TNP and the fluorescent quenching of HBAPB by *p*-NP. The flexible COFs have great potential for manufacturing sensors, and this potential deserves deep research.

## Figures and Tables

**Table 1:** The equations of I_0_/I of the FFCP COFs to the concentrations of TNP or *p*-NP for dispersions in ACN (HDADE), THF, (HBAPB) and DOX (HBPDA).

COFs	The Equations	Regression Coefficient (R)	Concentration Range of TNP or *p*-NP (mol L^−1^)	Limit of Detections (mol L^−1^)
HDADE	I_0_/I = 1.0016 + 6.29 × 10^4^ [TNP]	0.966	0 to 1.0 × 10^−5^	1.19 × 10^−11^
HBAPB	I_0_/I = 0.9883 + 2.17 × 10^5^ [*p*-NP]	0.9984	0 to 5.0 × 10^−5^	6.91 × 10^−12^
HBPDA	I_0_/I = 0.6245 + 2.48 × 10^5^ [*p*-NP]	0.9960	0 to 7.5 × 10^−5^	6.05 × 10^−12^

**Table 2:** Summary of Ksv and LODs of the COFs for fluorescence sensing to NACs.

Sample	NACs	K_sv_ (L mol^−1^)	LODs (mol L^−1^)	Refs
Py-Azine COF	TNP	7.6 × 10^4^	-	[24]
	*o*-NP	5.9 × 10^2^	-	
FL-SNW-DPP-0.11	TNP	5.3 × 10^4^	-	[25]
TfpBDH-CONs	TNP	2.6 × 10^4^	-	[26]
	*p*-NP	−		
TAT-COF-2	NB	−	10 × 10^–6^	[58]
TRIPTA	PA	2.7 × 10^6^	-	[59]
	*p*-NP	−	-	
3D-Py-COF	TNP	3.1 × 10^4^	-	[27]
COF-BABD-DB	TNP	5.7 × 10^5^	-	[28]
	*p*-NP	1.55 × 10^4^	-	
COF-BABD-BZ	TNP	4.5 × 10^5^	-	
	*p*-NP	3.2 × 10^4^	-	
PAF-130	TNP	4.69 × 10^4^	-	[57]
	*o*-NP	−	-	
HBD	*p*-NP	7.91 × 10^4^	4.6 × 10^−11^	[5]
HDASD	*p*-NP	1.16 × 10^5^	2.59 × 10^−10^	
HDOBD	*p*-NP	3.76 × 10^4^	7.98 × 10^−11^	
HDADE	TNP	6.29 × 10^4^	1.19 × 10^−11^	This work
HBAPB	*p*-NP	2.17 × 10^5^	6.91 × 10^−12^	
HBPDA	*p*-NP	2.48 × 10^5^	6.05 × 10^−12^	

## Supplementary Materials

The following supporting information can be downloaded at: https://www.mdpi.com/article/10.3390/polym15030653/s1. Scheme S1: Reaction equation for the building block NOP-6-CHO; Scheme S2: Synthesis routes of the fully flexible cyclotriphosphazene-based COFs. Figure S1: The FT-IR spectra of the fully flexible cyclophosphazene-based COFs and corresponding building blocks: (a) HDADE, (b) HBAPB, (c) HBPDA, (d) DADE, (e) BAPB, (f) BPDA and (f) NOP-6-CHO; Figure S2: The ss 13C NMR spectra of HDADE, HBAPB, and HBPDA. Figure S3: Solid state UV-Vis spectra of the fully flexible cyclophosphazene-based COFs and corresponding building blocks (a) HDADE, (b) HBAPB and (c) HBPDA. Figure S4: SEM images of the fully flexible cyclophosphazene-based COFs (A) HDADE, (B) HBAPB and (C) HBPDA (Scale 50 μm, the voltage 15.0 kV, magnification 33.6 mm × 3.00k SE). Figure S5: Experimental (black) and predicted (red) powder X-ray diffraction (PXRD) patterns of the fully flexible cyclophosphazene-based COFs: (a) HDADE, (b) HBAPB and (c) HBPDA samples (inset: views of space-filling models along the c-axis with the layer distances). Figure S6: (a) Nitrogen adsorption-desorption isotherms of HDADE, HBAPB, and HBPDA (adsorption-solid square, desorption-empty circle). (b) Pore size distribution of the three fully flexible cyclophosphazene-based COFs. Table S1: Specific surface areas, pore valumes, and pore size distributions of FFCP COFs. Figure S7: PXRD patterns of the COFs (a) HDADE, (b) HBAPB and (c) HBPDA before and after soaked in different solvents for two days. Figure S8: Thermogravimetric analysis (TGA) images of HDADE, HBAPB, and HBPDA in the nitrogen atmosphere with the heating rates of 10 °C min^−1^. Figure S9: Thermogravimetric analysis (TGA) images of HDADE, HBAPB, and HBPDA in the air atmosphere with the heating rates of 10 °C min^−1^. Table S2: HOMO and LUMO calculations for the fully flexible cyclotriphosphazene-based COFs and NACs. All the molecular orbital calculations were performed with the Gaussian 09 D.01 program at the B3LYP/6-31G* level. Figure S10: Cycling tests of the fully flexible cyclotriphosphazene-based COFs dispersed in ACN (HDADE, TNP: 7.5 × 10^−5^ mol L^−1^, λex = 248 nm), THF (HBAPB, *p*-NP: 5.0 × 10^−5^ mol L^−1^, λex = 305 nm) and DOX (HBPDA, *p*-NP: 7.5 × 10^−5^ mol L^−1^, λex = 311 nm) (1.0 mg mL^−1^). Figure S11: FT-IR spectra of (a) HDADE, (b) HBAPB, and (c) HBPDA before (black line) and after (red line) application. Figure S12: PXRD patterns of (a) HDADE, (b) HBAPB, and (c) HBPDA before (black line) and after (red line) application.
