# Supplementary material for: Fully Flexible Covalent Organic Frameworks for Fluorescence Sensing 2,4,6-Trinitrophenol and p-Nitrophenol
Source: Polymers (Basel). 2023 Jan 27;15(3):653. doi: 10.3390/polym15030653 (PMC9919289; doi:10.3390/polym15030653)
Supplement: Supplementary file 1 [file polymers-15-00653-s001.zip › polymers-2117896-supplementary.pdf]

Supplementary materials

for

Fully flexible Covalent Organic Frameworks for Fluorescence Sensing 2,4,6-Trinitrophenol and *p*–Nitrophenol

Hai Zhu [1,2], Tong-Mou Geng [2,*], Kai-Bin Tang [1,*]

1 Department of Chemistry, University of Science and Technology of China, Hefei 230026, People's Republic of China

2 AnHui Province Key Laboratory of Optoelectronic and Magnetism Functional Materials; School of Chemistry and Chemical Engineering, Anqing Normal University, Anqing 246011, China

Corresponding Author:

Tong-Mou Geng

Mailing Address: School of Chemistry and Chemical Engineering, Anqing Normal University, Anqing 246011, China

E-mail addresses: gengtongmou@aqnu.edu.cn (TM Geng).

Kai-Bin Tang

Mailing Address: Department of Chemistry, University of Science and Technology of China, Hefei 230026, People's Republic of China.

E-mail addresses: kbtang@ustc.edu.cn (KB Tang).

S1 Experimental section

S1-1 Materials and general procedures

S1-1-1 Chemical materials

4,4′-Diaminodiphenyl ether (DADE), 1,4-bis(4-aminophenoxy)benzene (BAPB), 4,4′-(1,1′-biphenyl-4,4′-diyldioxy)dianiline (BPDA), triethylamine, and 4-hydrobenzaldehyde were purchased from Aladdin chemical reagent Limited (Shanghai). Hexachlorocyclotriphosphazene (HCCP) was obtained from Shanghai Shaoyuan reagent Co., Ltd. Other starting materials, reagents and solvents were purchased from commercial sources and used without further purification.

S1-2 Synthesis of a building block and COFs

S1-2-1. Synthesis of hexa(4-formyl-phenoxy)cyclotriphosphazene (NOP-6-CHO)

NOP–6–CHO was synthesized according to a previous synthetic report with minor modifications. The appropriate amount of triethylamine (16.5 mL, 0.119 mol) p-hydroxybenzaldehyde (11.1 g, 0.091 mol), hexachlorocyclotriphosphazene (HCCP) (5.214 g, 0.015 mol), and 80 mL of tetrahydrofuran (THF) were added to a three-neck flask equipped with mechanical stirring, thermometer, and condenser, and reacted 30 min under stirring conditions. After 2 hours of ice bath, stirring reaction was conducted at 80 ^{o}C for 2 days. After completion of the reaction, the mixture was cooled to room temperature and filtered. The filtrate was concentrated under reduced pressure. The concentrated residue was then recrystallized from ethyl acetate yielding a yellow powder of 1 (9.7253 g, 76.99 % yield) [S1,S2]. Elemental analysis: theoretical values: C 58.84, H 3.90, N 4.79; measured values: C 58.38, H 3.66, N 4.

937.

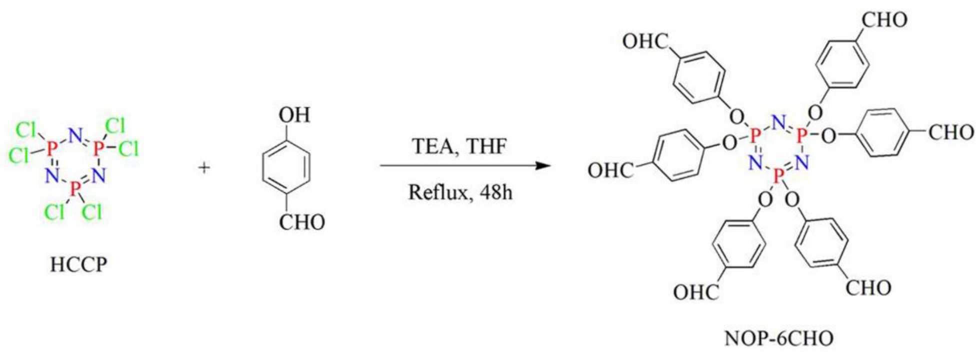

Scheme S1. Reaction equation for the building block NOP–6–CHO.

Reference

S1. Das, G.; Prakasam, T.; Nuryyeva, S.; Han, D.S.; Wahab, A.A.; Olsen, J.C.; Polychronopoulou, K.; Platas-Iglesias, C.; Ravaux, F.; Jouiad, M.; Trabolsi, A. Multifunctional redox-tuned viologen-based covalent organic polymers. J. Mater. Chem. A 2016, 4, 15361–15369.

S2. Guo, X.H.; Li, Y.; Zhang, M.C.; Cao, K.C.; Tian, Y.; Qi, Y.; Li, S.J.; Li, K.; Yu, X.Q.; Ma, L.J. Colyliform crystalline 2D covalent organic frameworks with quasi-3D topologies for rapid I_2 adsorption, Angew. Chem. 2020, 50, 22886–22894.

S1-2-2 Synthesis of HDADE

NOP–6–CHO (0.7021 g, 0.8 mmol) and DADE (0.4806 g, 2.4 mmol) were placed in 100 mL pressure vessel respectively, then 1,2-dichlorobenzene/*n*-butanol/6 mol L^{-1} acetic acid (5:5:1 v/v/v, 44 mL) was added and the two mixtures were sonicated for 5 min to afford homogeneous dispersion and protected by nitrogen, The vessel was then sealed and left undisturbed for 3 days at 120 °C. The solid was

collected by filtration and washed with acetone and THF separately. The powder was dried at 50 ºC under vacuum overnight to afford khaki powder in 71.73 % (0.7863 g) isolated yield and elemental analysis. ss ^{13}C NMR (δ ppm): 153.69, 144.84, 133.94, 122.61. Elemental analysis: (%) calc/found: C 68.47/68.63, H 5.16/4.007, N 6.76/6.63.

S1-2-3 Synthesis of HBAPB

NOP–6–CHO (0.7021 g, 0.8 mmol) and BAPB (0.7016 g, 2.4 mmol) were placed in 100 mL pressure vessel respectively, then 1,2-dichlorobenzene/*n*-butanol/6 mol L^{-1} acetic acid (5:5:1 v/v/v, 44 mL) was added and the two mixtures were sonicated for 5 min to afford homogeneous dispersion and protected by nitrogen. The vessel was then sealed and left undisturbed for 3 days at 120 °C. The solid was collected by filtration and washed with acetone and THF separately. The powder was dried at 50 ºC under vacuum overnight to afford a khaki powder in 72.14 % (0.9502 g) isolated yield and elemental analysis. ss ^{13}C NMR (δ ppm): 152.24, 144.30, 133.94, 121.59. Elemental analysis: (%) calc/found: C 70.53/70.76, H 5.00/4.150, N 6.41/6.59.

S1-2-4 Synthesis of HBPDA

NOP–6–CHO (0.7021 g, 0.8 mmol) and BPDA (0.8842 g, 2.4 mmol) were placed in 100 mL pressure vessel respectively, then 1,2-dichlorobenzene/*n*-butanol/6 mol L^{-1} acetic acid (5:5:1 v/v/v, 44 mL) was added and the two mixtures were sonicated for 5 min to afford homogeneous dispersion and protected by nitrogen, The vessel was then sealed and left undisturbed for 3 days at 120 °C. The solids were

collected by filtration and washed with acetone and THF separately. The powder was dried at 50 ºC under vacuum overnight to afford a beige yellow solid in 73.07 % (1.1141 g) isolated yield and elemental analysis. ss ^{13}C NMR (δ ppm): 155.68, 143.33, 130.99, 127.01, 120.62. Elemental analysis: (%) calc/found: C 74.01/74.10, H 4.74/4.230, N 5.57/5.34.

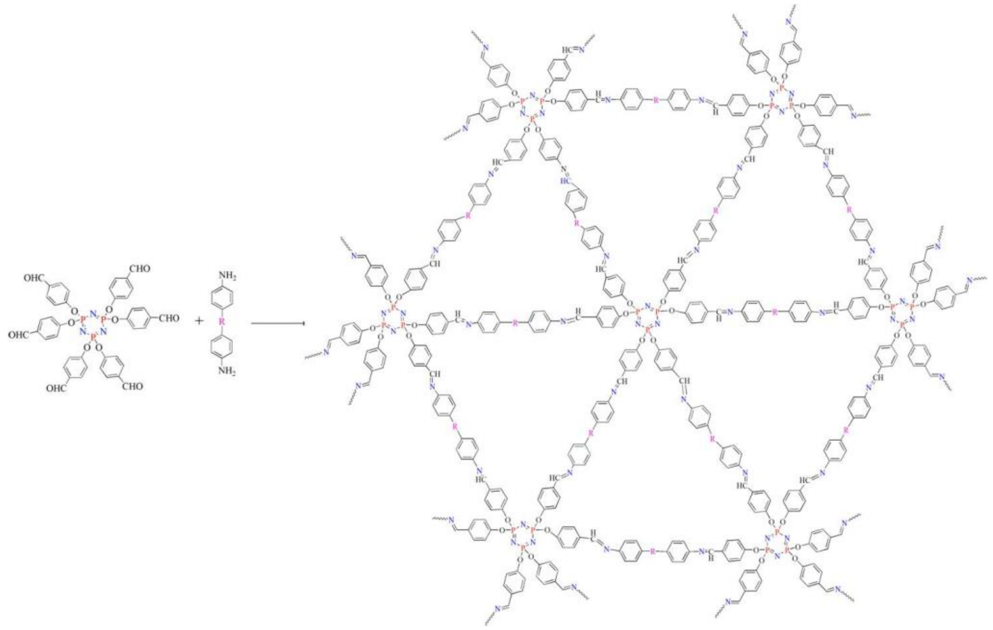

Scheme S2. Synthesis routes of the fully flexible cyclotriphosphazene-based COFs: R = O: HDADE, R = 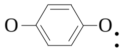: HBAPB, and R = 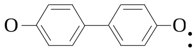: HBPDA.

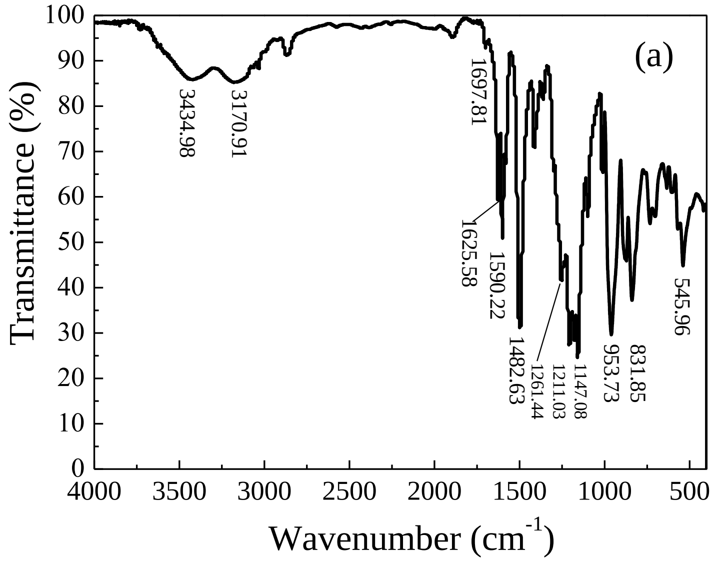

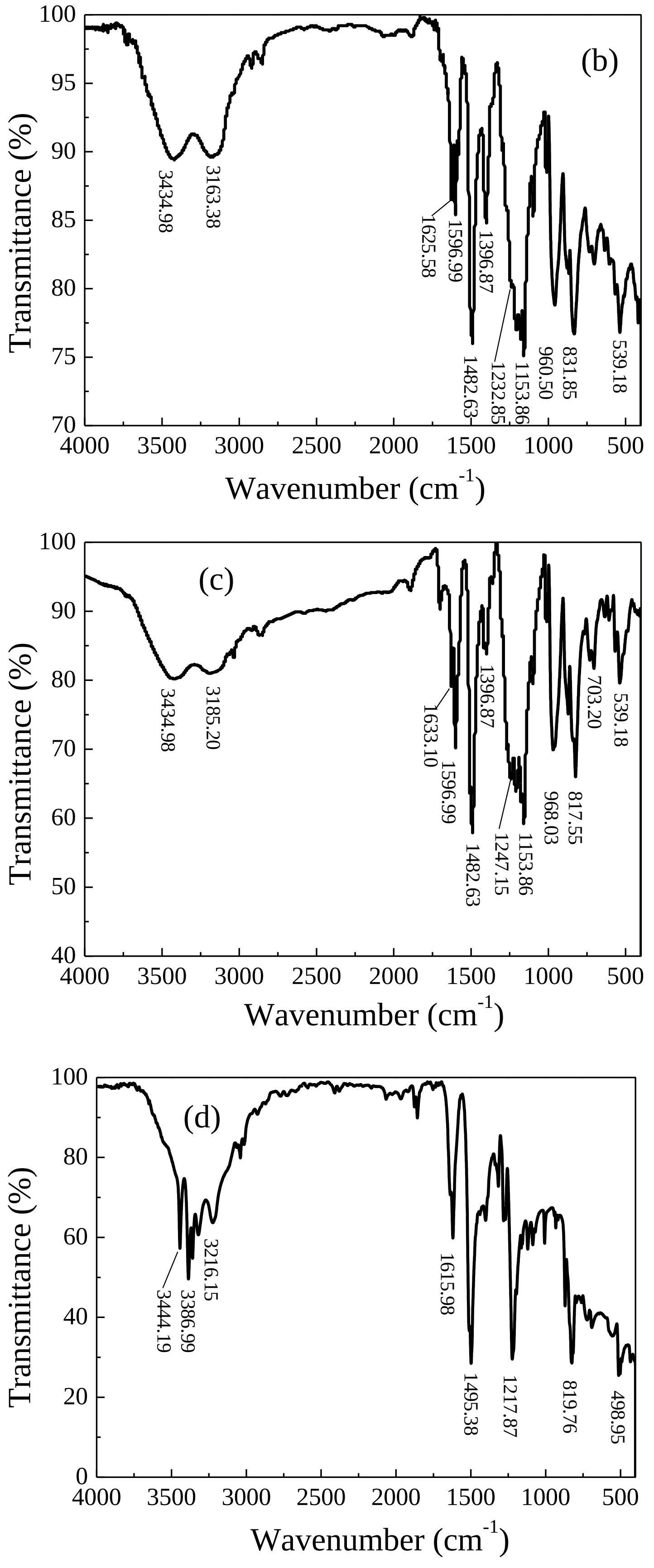
(b)
Transmittance (%)
Wavenumber (cm-1)
3434.98
3163.38
1625.58
1596.99
1396.87
1482.63
1232.85
1153.86
960.50
831.85
539.18
(c)
Transmittance (%)
Wavenumber (cm-1)
3434.98
3185.20
1633.10
1596.99
1396.87
1482.63
1247.15
1153.86
968.03
817.55
703.20
539.18
(d)
Transmittance (%)
Wavenumber (cm-1)
3444.19
3386.99
3216.15
1615.98
1495.38
1217.87
819.76
498.95

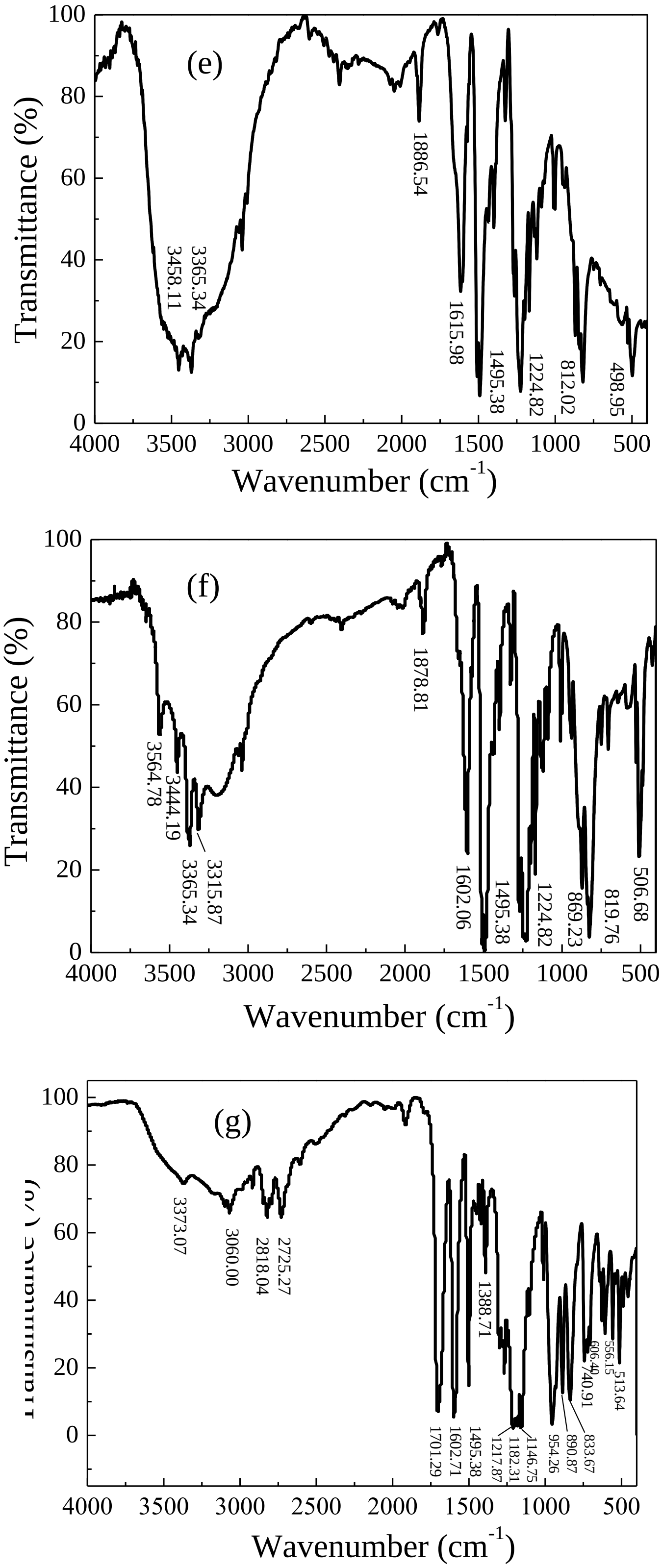

Figure S1. The FT-IR spectra of the fully flexible cyclophosphazene-based COFs and corresponding building blocks: (a) HDADE, (b) HBAPB, (c) HBPDA, (d) DADE, (e) BAPB, (f) BPDA and (f) NOP–6–CHO.

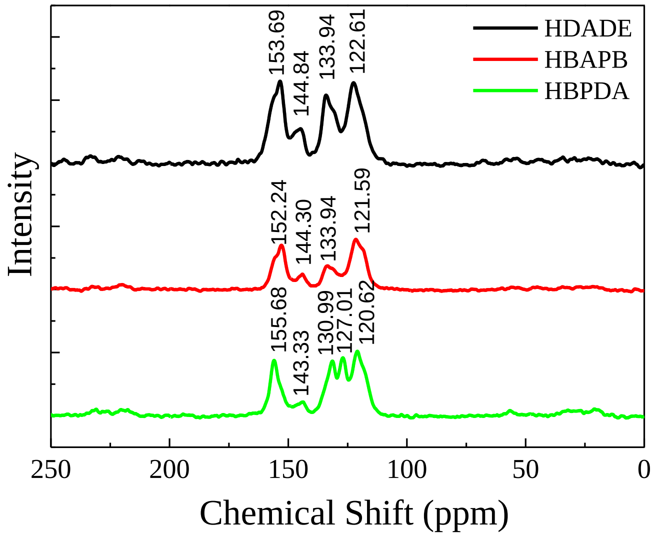

Figure S2. The ss ^{13}C NMR spectra of HDADE, HBAPB, and HBPDA.

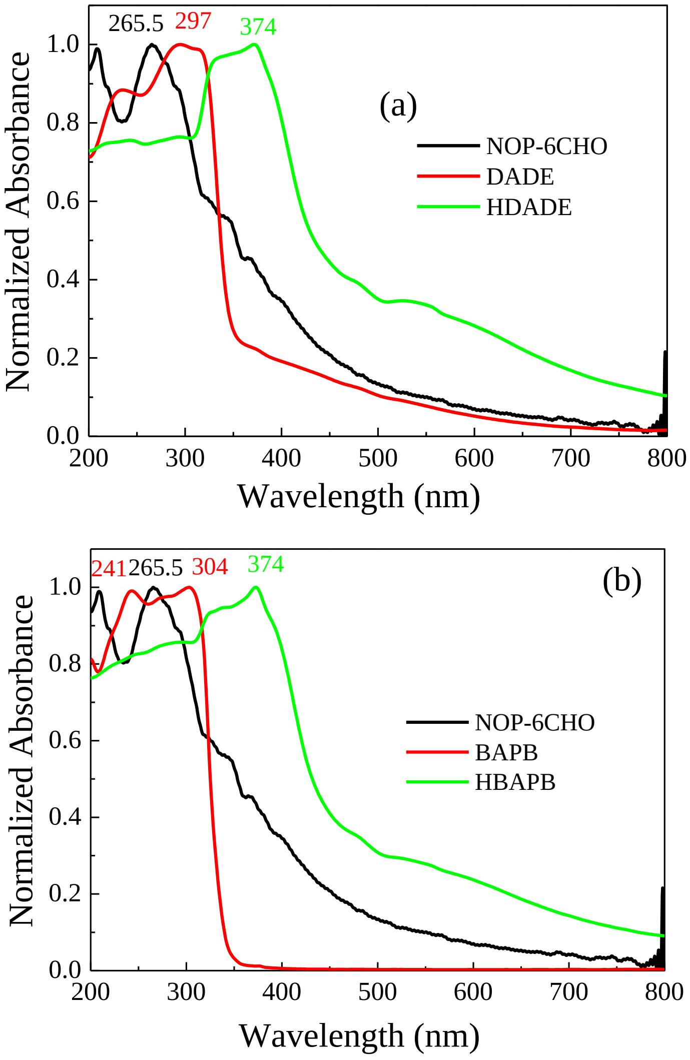

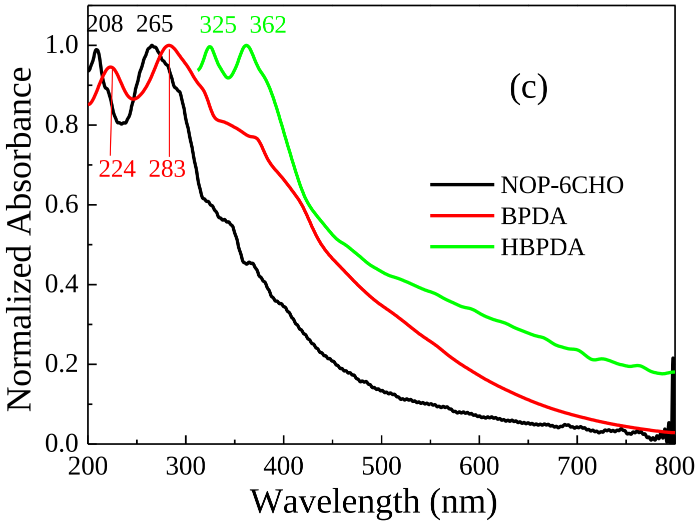

Figure S3. Solid state UV-Vis spectra of the fully flexible cyclophosphazene-based COFs and corresponding building blocks (a) HDADE, (b) HBAPB and (c) HBPDA.

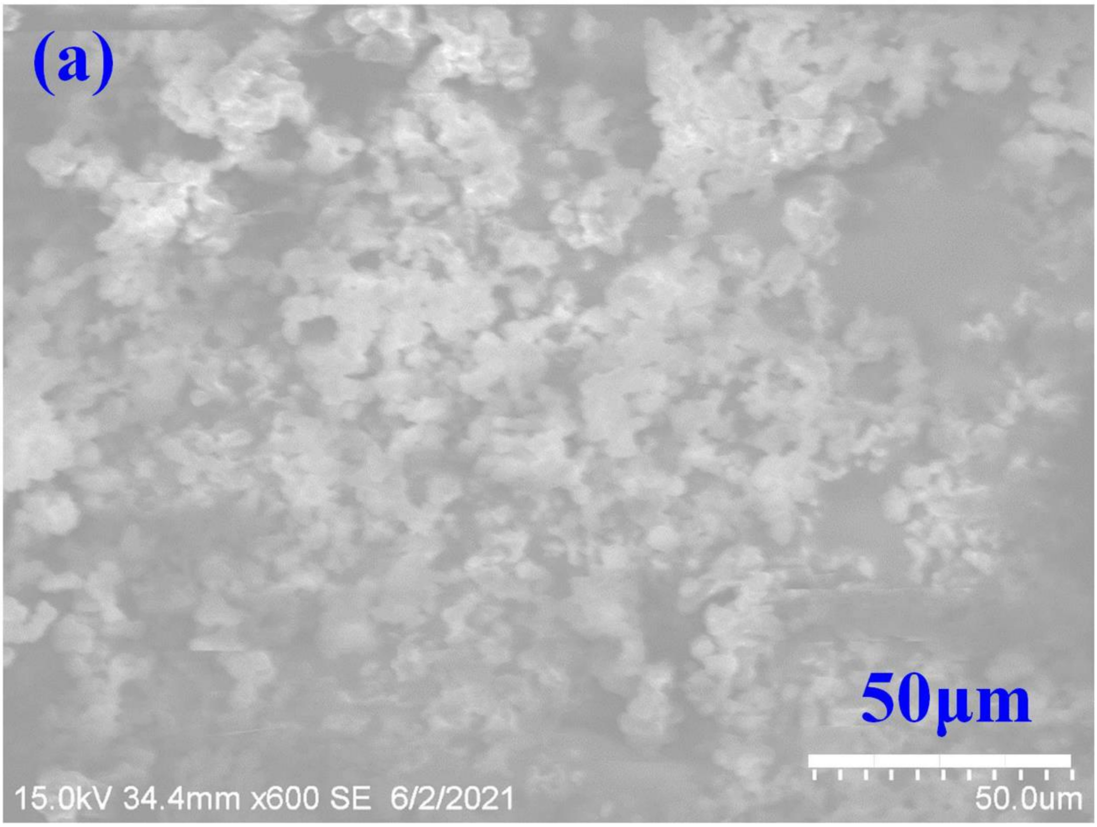

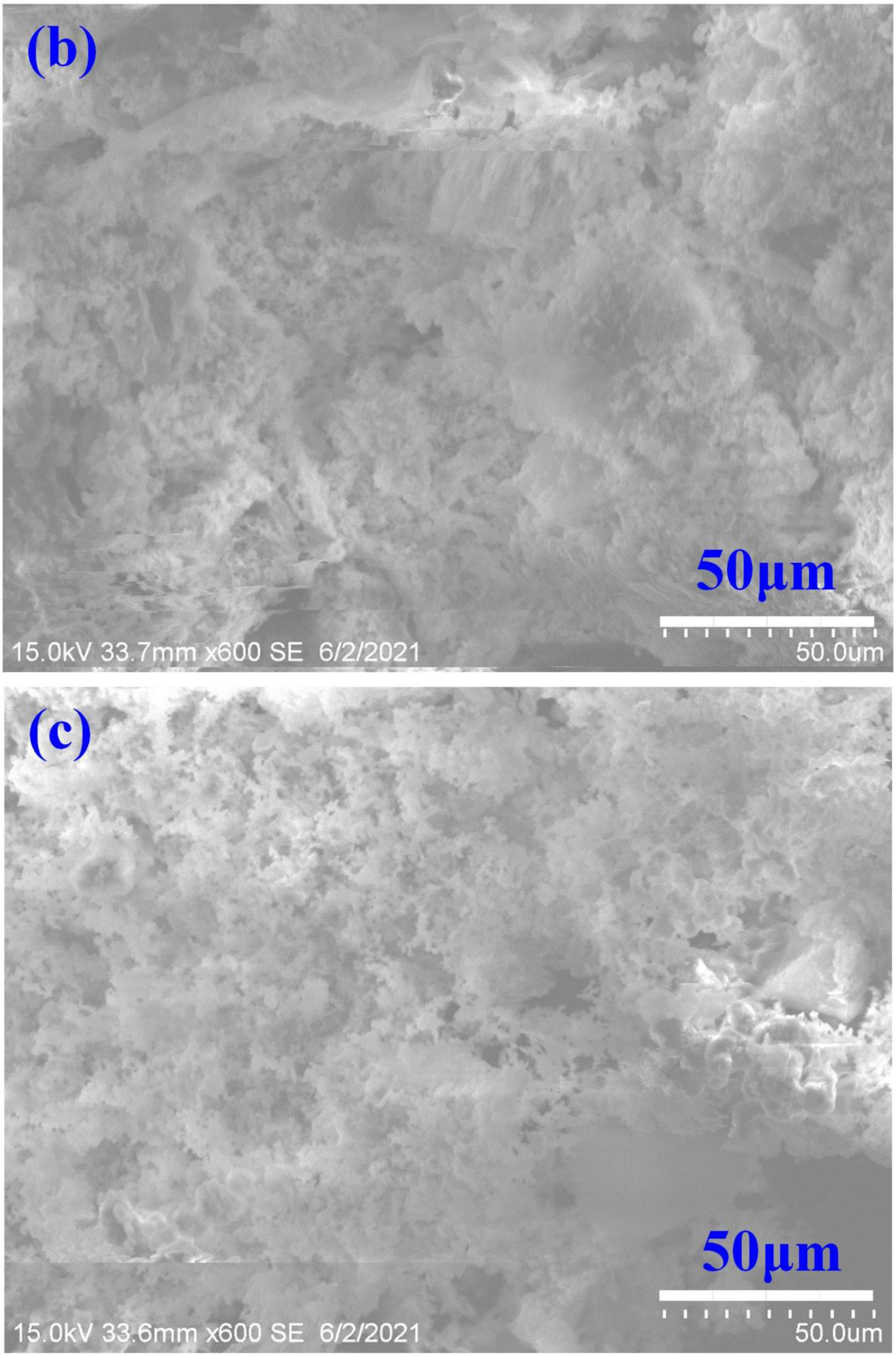

Figure S4. SEM images of the fully flexible cyclophosphazene-based COFs (A) HDADE, (B) HBAPB and (C) HBPDA (Scale 50 μm, the voltage 15.0 kV, magnification 33.6 mm×3.00k SE).

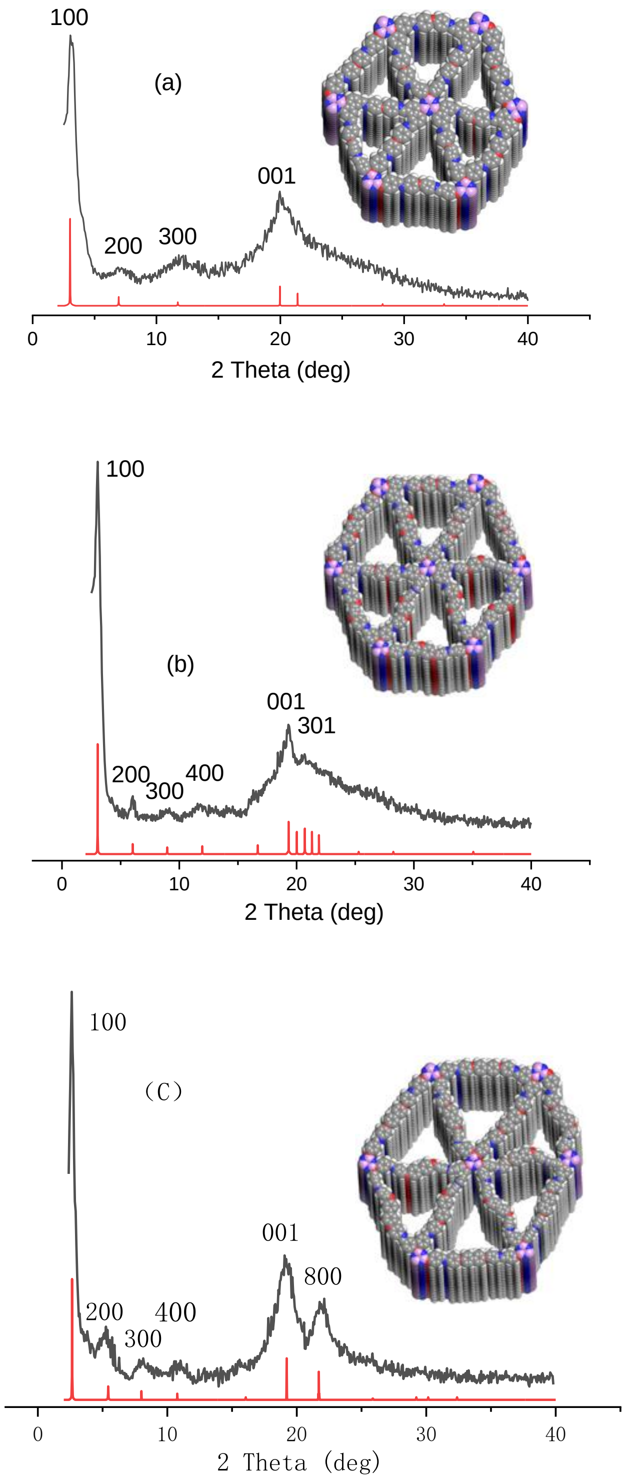

Figure S5. Experimental (black) and predicted (red) powder X-ray diffraction (PXRD) patterns of the fully flexible cyclophosphazene-based COFs: (a) HDADE, (b) HBAPB and (c) HBPDA samples (inset: views of space-filling models along the c-axis with the layer distances).

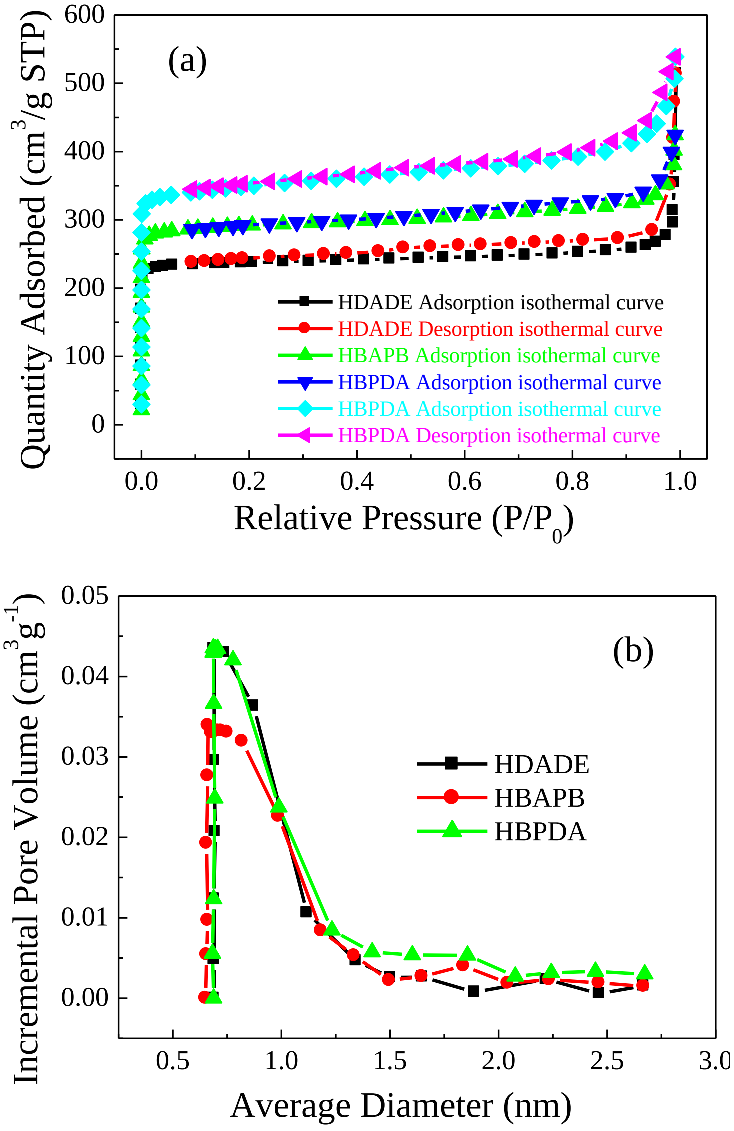

Figure S6. (a) Nitrogen adsorption–desorption isotherms of HDADE, HBAPB, and HBPDA (adsorption–solid square, desorption–empty circle). (b) Pore size distribution of the three fully flexible cyclophosphazene-based COFs.

Table S1. Specific surface areas, pore valumes, and pore size distributions of FFCP COFs.

FFCP COFs	S_{BET}[a] ($m^2 g^{-1}$)	$S_{Langmuir}$[a] ($m^2 g^{-1}$)	V_{total} (tpv)[b] ($cm^3 g^{-1}$)	V_{micro}[c] ($cm^3 g^{-1}$)	Main pore diameter (nm)	Average pore diameter (nm)

HDADE	790	1039	0.7949	0.3677	0.690	4.03
HBAPB	975	1283	0. 6564	0.4427	0.691	2.69
HBPDA	1165	1541	0.8334	0.5337	0.688	2.86

a Specific surface area is calculated from the adsorption branch of the nitrogen isotherm using the BET method in the relative pressure (P/P_0) range from 0.01 to 0.10.

b Total pore volume is obtained from BET data up to P/P_0=0.97 and is defined as the sum of micropore volume and volumes of larger pores.

c Micropore volume calculated from nitrogen adsorption isotherm using the t-plot method.

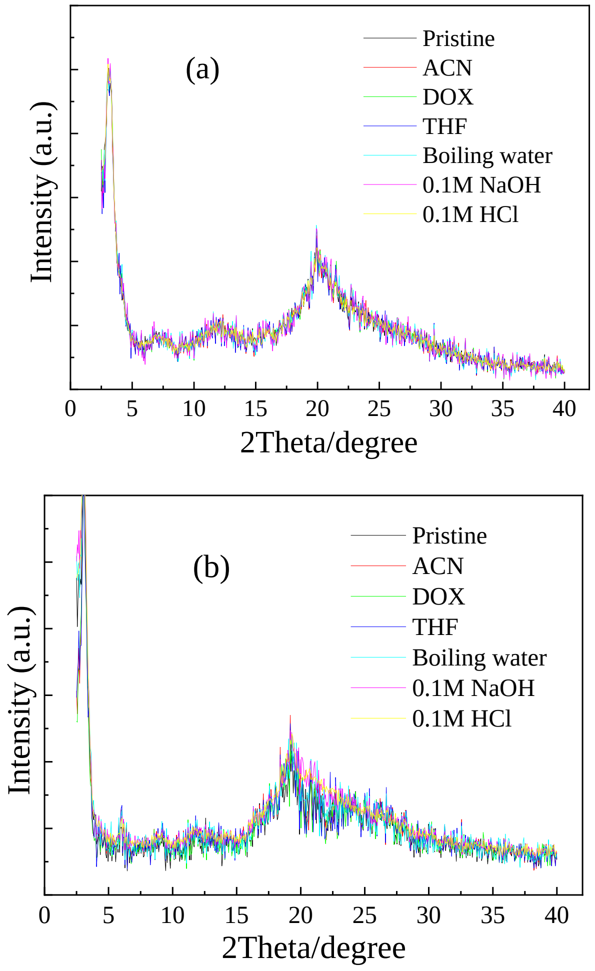

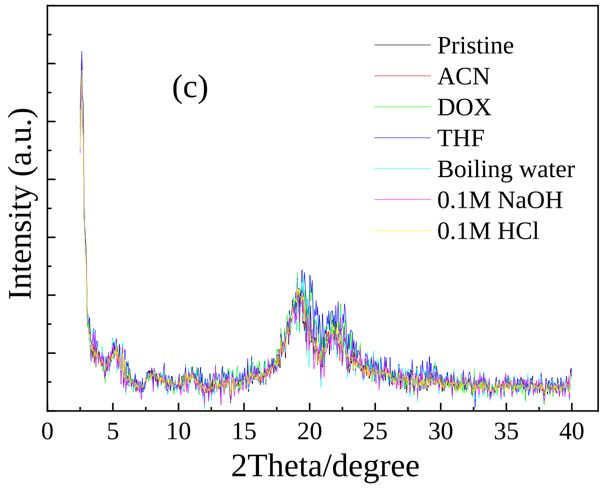

Figure S7. PXRD patterns of the COFs (a) HDADE, (b) HBAPB and (c) HBPDA before and after soaked in different solvents for two days.

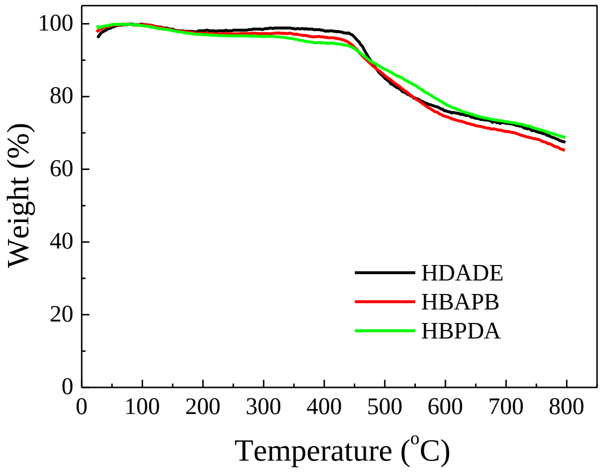

Figure S8. Thermogravimetric analysis (TGA) images of HDADE, HBAPB, and HBPDA in the nitrogen atmosphere with the heating rates of 10 °C min^{-1}.

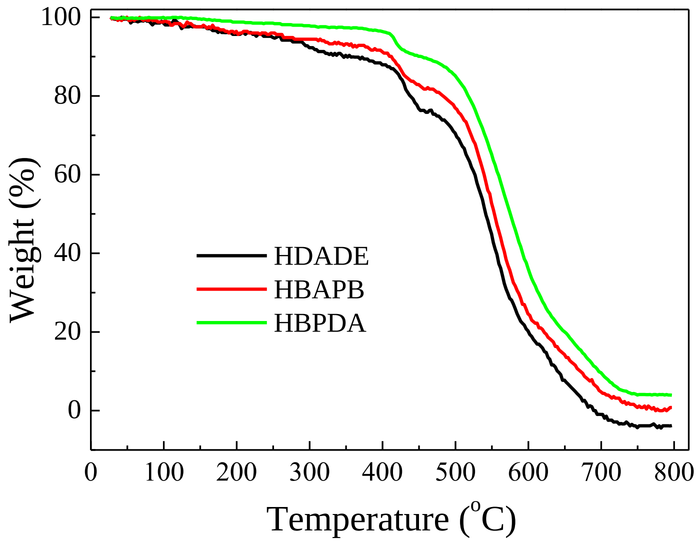

Figure S9. Thermogravimetric analysis (TGA) images of HDADE, HBAPB, and HBPDA in the air atmosphere with the heating rates of 10 °C min^{-1}.

Table S2. HOMO and LUMO calculations for the fully flexible cyclotriphosphazene-based COFs and NACs. All the molecular orbital calculations were performed with the Gaussian 09 D.01 program at the B3LYP/6-31G* level

MO energy (eV)	HDADE	HBAPB	HBPDA	TNP	DNP
LUMO	-1.665	-1.673	-1.695	-3.898	-3.320
HOMO	-5.016	-5.438	-5.404	-8.273	-7.628
MO energy (eV)	o-NP	m-NP	*p*−NP	NB	m-DNB
LUMO	-2.711	-2.396	-2.222	-2.428	-3.135
HOMO	-6.797	-6.779	-6.721	-7.591	-8.413
MO energy (eV)	p-DNB	DNT	*p*−NT		
LUMO	-3.495	-2.977	-2.318		
HOMO	-8.358	-8.113	-7.364		

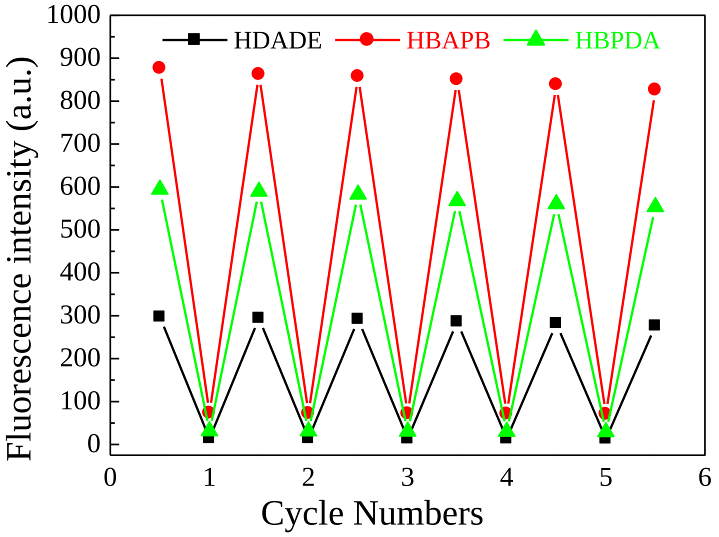

Figure S10. Cycling tests of the fully flexible cyclotriphosphazene-based COFs dispersed in ACN (HDADE, TNP: 7.5×10^{-5} mol L^{-1}, λex=248 nm), THF (HBAPB, *p*–NP: 5.0×10^{-5} mol L^{-1}, λex=305 nm) and DOX (HBPDA, *p*–NP: 7.5×10^{-5} mol L^{-1}, λex=311 nm) (1.0 mg mL^{-1}).

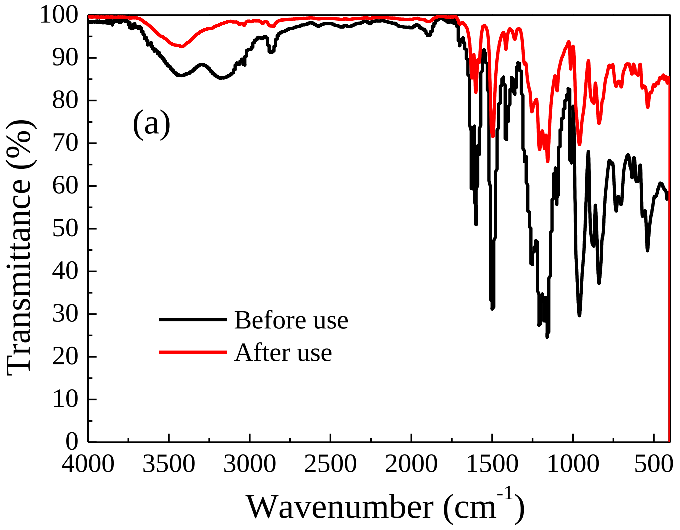

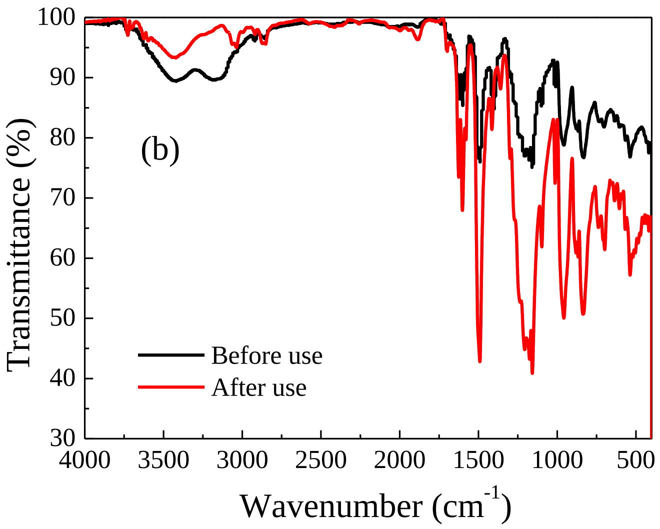

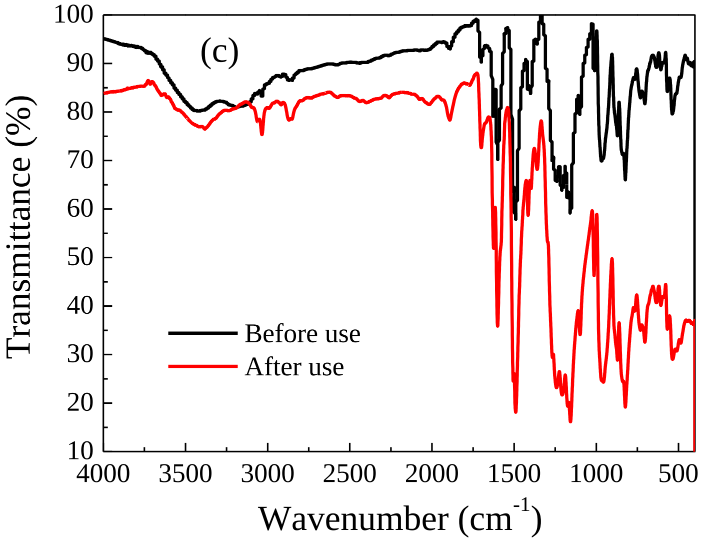

Figure S11. FT-IR spectra of (a) HDADE, (b) HBAPB, and (c) HBPDA before (black line) and after (red line) application.

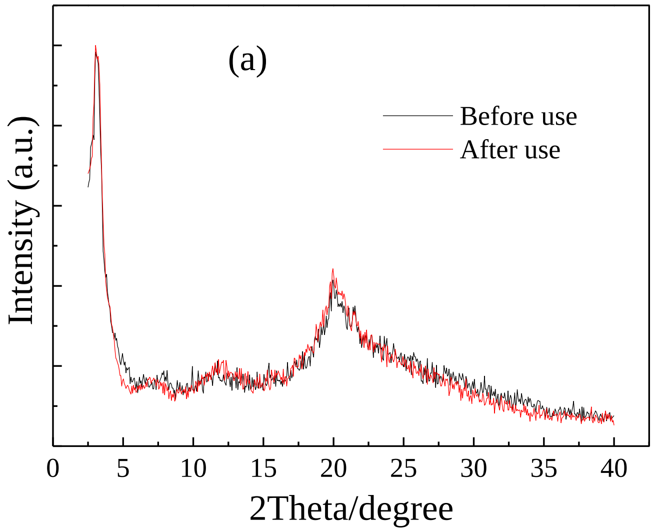

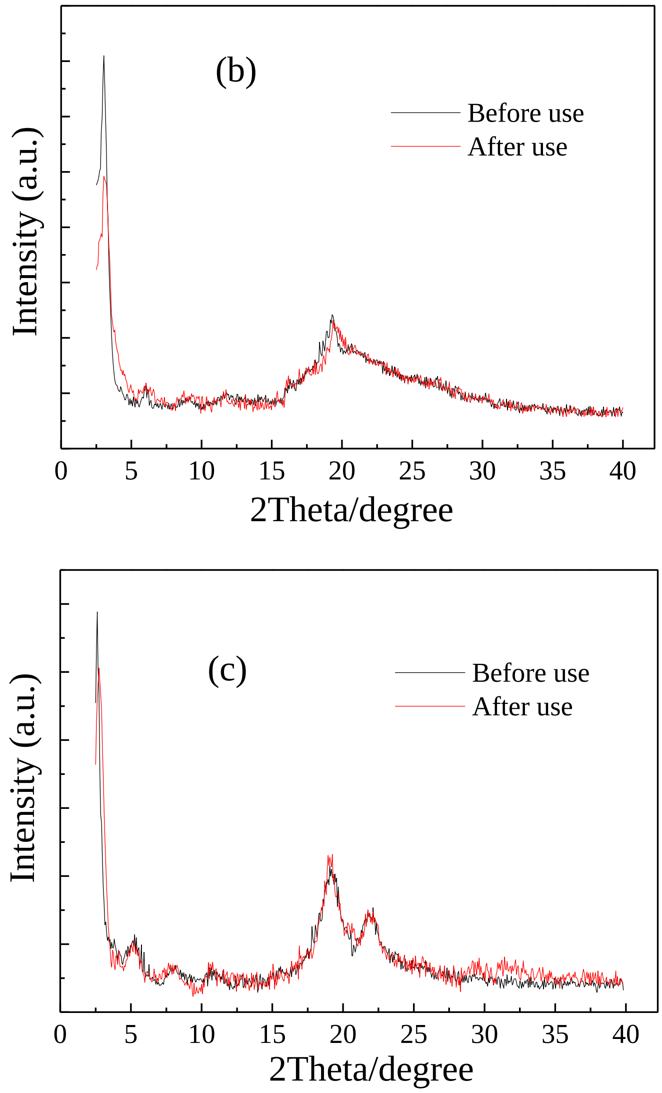

Figure S12. PXRD patterns of (a) HDADE, (b) HBAPB, and (c) HBPDA before (black line) and after (red line) application.
